# Short Stature: Comparison of WHO and National Growth Standards/References for Height

**DOI:** 10.1371/journal.pone.0157277

**Published:** 2016-06-09

**Authors:** Henrik Thybo Christesen, Birgitte Tønnes Pedersen, Effie Pournara, Isabelle Oliver Petit, Pétur Benedikt Júlíusson

**Affiliations:** 1 Hans Christian Andersen Children’s Hospital, Odense University Hospital, Odense, Denmark; 2 Epidemiology, Novo Nordisk A/S, Søborg, Denmark; 3 Novo Nordisk Health Care AG, Zurich, Switzerland; 4 Department of Paediatric Endocrinology, Hôpital des Enfants, Toulouse, France; 5 Department of Clinical Science, University of Bergen, Bergen, Norway; 6 Department of Pediatrics, Haukeland University Hospital, Bergen, Norway; UNAIDS, GUYANA

## Abstract

The use of appropriate growth standards/references is of significant clinical importance in assessing the height of children with short stature as it may determine eligibility for appropriate therapy. The aim of this study was to determine the impact of using World Health Organization (WHO) instead of national growth standards/references on height assessment in short children. Data were collected from routine clinical practice (1998–2014) from nine European countries that have available national growth references and were enrolled in NordiNet^®^ International Outcome Study (IOS) (NCT00960128), a large-scale, non-interventional, multinational study. The patient cohort consisted of 5996 short pediatric patients diagnosed with growth hormone deficiency (GHD), Turner syndrome (TS) or born small for gestational age (SGA). The proportions of children with baseline height standard deviation score (SDS) below clinical cut-off values (–2 SDS for GHD and TS; –2.5 SDS for SGA) based on national growth references and WHO growth standards/references were compared for children aged <5 years and children aged ≥5 years. In seven of the countries evaluated, significantly fewer children aged ≥5 years with GHD (22%; *P*<0.0001), TS (21%; *P*<0.0001) or born SGA (32%; *P*<0.0001) had height below clinical cut-off values using WHO growth references vs. national references. Likewise, among children aged <5 years in the pooled analysis of the same seven countries, a significantly lower proportion of children with GHD (8%; *P*<0.0001), TS (12%; *P* = 0.0003) or born SGA (12%; *P*<0.0001) had height below clinical cut-off values using WHO growth standards vs. national references. In conclusion, in NordiNet^®^ IOS the number of patients misclassified using WHO growth standards/references was significantly higher than with national references. This study highlights that, although no growth reference has 100% sensitivity for identifying growth disorders, the most recent national or regional growth charts may offer the most appropriate tool for monitoring childhood growth in Europe.

## Introduction

Childhood growth and development is routinely monitored using length/height, weight and head circumference [1]. Abnormal growth patterns may suggest underlying medical or social conditions requiring further investigation [1]. Deviations from normal growth patterns are assessed by comparing actual height values with appropriate age- and sex-specific growth references, which are universally considered by pediatricians as a crucial tool for correct diagnosis and timely intervention in many medical conditions [2, 3]. The evaluation of childhood growth is dependent on the growth charts used, with the recently updated references based on the population under evaluation providing the most accurate reflection of a population’s height. Secular trends showing incremental increases in growth result in increased average height over generations [4, 5], especially in affluent populations in which socioeconomic constraints on growth are minimized [6, 7]. Thus, national growth references are likely to become outdated in the years after construction, although the secular trend in length/height may be levelling off in some countries [8]. Consequently, regular updates to all growth references are required to allow more accurate screening for height disorders [5].

International growth references may be more appropriate than national references in light of population migration between countries. An early international reference came from the International Children's Center study, conducted in London, Paris, Zürich, Stockholm, Brussels, Louisville (KY) and Dakar from 1953 onwards, of which the data from Paris are still being used as growth reference in France [9]. In 2006, multi-ethnic growth standards for children aged <5 years were released by the World Health Organization (WHO) compiled from the Multicentre Growth Reference Study, based on growth data collected between 1997 and 2003 from 6669 economically advantaged, breastfed infants and children of non-smoking mothers from six countries [10]. The premise for constructing these growth standards was that unconstrained growth does not vary substantially and therefore one growth curve would be appropriate to describe normal growth [10]. WHO describes its growth curves as growth standards, providing normal targets for children’s growth in all countries [11]. These standards differ from growth references for which the aim is to show how healthy children actually grow in a given time and place. In 2007, WHO published a reference for children and adolescents aged 5–19 years by reconstructing the National Center for Health Statistics/WHO growth reference data from 1977 [12]. By 2010, over 100 countries had adopted WHO standards or references [13].

Accumulating evidence suggests that the height ranges of healthy children aged 0–5 years in many countries, including Belgium, Canada, Denmark, Norway and Turkey, differ from the normal ranges according to WHO growth standards [6, 14–20]. The WHO growth standards provide lower cut-offs for short stature than many of these references, which are generally newer. As a consequence, the assumption that universal WHO growth standards can be used globally to accurately screen for linear growth disorders is challenged.

Moreover, the WHO growth references for children aged 5–19 years are based on re-analyzed data from the USA between 1960 and 1980, and may also provide lower cut-offs due to the age of the data and because children in the USA historically have been shorter than their European counterparts.

The aim of the present analysis was therefore to compare potential differences between the WHO growth standards/references and available national growth references in the Czech Republic, Denmark, France, Germany, Netherlands, Norway, Sweden, Switzerland and the UK on height assessment in children with short stature enrolled in NordiNet^®^ International Outcome Study (IOS). NordiNet^®^ IOS (NCT00960128) is a large-scale, non-interventional, observational study designed to gather long-term data on the safety and effectiveness of Norditropin^®^ (recombinant human growth hormone [GH], somatropin; Novo Nordisk A/S, Denmark) as prescribed by treating physicians in everyday clinical practice, and to provide insight into the diseases of the specific endocrine patient populations treated with GH [21]. The implications of using various references and standards in clinical contexts are discussed.

## Materials and Methods

### Ethics statement

NordiNet^®^ IOS is conducted in accordance with the Declaration of Helsinki; all patients provide written informed consent for data collection. All data collected within NordiNet^®^ IOS are anonymized. Approval of the local ethics committee or institutional review board at the individual center level in accordance with country-specific rules is a prerequisite for each center’s inclusion in NordiNet^®^ IOS. The data are completely anonymized prior to access for analysis. It is mandatory for the parents or guardians of the pediatric patients to provide written informed consent for the minors’ data to be collected.

### Patient population

The patient cohort comprised 5996 pediatric patients with short stature, diagnosed with GHD, TS or SGA who were enrolled in NordiNet^®^ IOS and started GH treatment between 1989 and 2014. The majority of patients started GH treatment in the recent years of the study. The median date for enrollment in NordiNet^®^ IOS was 2008 for patients aged ≥5 years and 2009 for patients aged <5 years. Clinical diagnosis was based on the investigator’s decision and the International Classification of Diseases 10^th^ Revision (ICD-10) criteria. Note that patients with acquired GHD were not included in the analyses, because these children might still have had normal height at diagnosis [22].

### Study design

In this report we evaluate baseline height data from pediatric patients enrolled in nine of the 23 countries participating in NordiNet^®^ IOS that have available national growth references: Czech Republic, Denmark, France, Germany, Netherlands, Norway, Sweden, Switzerland and UK. Eligible patients for the analysis were already on, or about to start, treatment with Norditropin^®^ and had baseline height data.

### Statistical analysis

Data are presented as means with standard deviation (SD) and percentages. Height standard deviation scores (SDS) at GH treatment start were calculated using the most recent national growth chart references (Czech Republic [23]; Denmark [6]; France [9]; Germany [24]; Netherlands [7]; Norway [25]; Sweden [26]; Switzerland [27]; UK [28] and WHO growth standards [10] or WHO references [12]).

The proportions of children aged ≥5 and <5 years with heights below clinical cut-off values (height below –2 SDS for GHD and TS; –2.5 SDS for children born SGA) were calculated on country level based on national growth references and WHO growth standards/references. The difference in the proportion of patients classified with short stature (sensitivity) according to national or WHO growth standards/references was calculated by the following equation:

$$difference\;in\;sensitivity=\frac{n_{n}-n_{w}}{N}$$

where n_n_ = number of children with height below clinical cut-off values (national reference); n_w_ = number of children with height below clinical cut-off values (WHO reference); N = total.

The equality of the proportions classified with short stature according to national or WHO growth references was evaluated at country level using the McNemar test for the GHD, TS and SGA population of children aged ≥5 years. Due to the low number of patients aged <5 years in most of the countries, the McNemar test could not be applied at country level for all countries in this population. Hence, the McNemar test was also performed on pooled data of the countries that had an overall higher short stature classification rate based on the national growth references than with the WHO growth standards. For consistency a pooled analysis on patients aged ≥5 years was also applied.

## Results

Of the total cohort, 3593 (60%) patients were diagnosed with GHD, 1590 (26%) with short stature born SGA and 813 (14%) with TS. In both children aged <5 years and ≥5 years, a higher proportion of boys than girls was observed among children with GHD, but not among short children born SGA (Table 1). For children aged ≥5 years, age at treatment start was highest for patients with GHD and lowest for short children born SGA, and in the group aged <5 years, children with GHD had a lower mean age at GH start than those with TS or born SGA (Table 1).

**Table 1:** Baseline characteristics of for patients aged ≥5 and <5 years by indication.

	Children with GHD	Children with TS	Children born SGA
**Children aged ≥5 years, n (%)**			
Female	969 (33)	647 (100)	538 (47)
Male	1937 (67)	−	596 (53)
**Mean age at treatment start, years (SD)**			
Female	10.0 (2.7)	9.8 (3.1)	8.7 (2.6)
Male	10.4 (3.3)	–	8.9 (3.0)
**Children aged <5 years, n (%)**			
Female	241 (35)	166 (100)	237 (52)
Male	446 (65)	−	219 (48)
**Mean age at treatment start, years (SD)**			
Female	3.3 (1.2)	3.6 (1.0)	4.0 (0.8)
Male	3.5 (1.2)	–	4.1 (0.7)

GHD, growth hormone deficiency; SD, standard deviation; SGA, small for gestational age; TS, Turner syndrome.

### Children aged ≥5 years

With the exception of France and the UK, proportionally more children aged ≥5 years across all three indications by country had height SDS below clinical cut-off values using national growth references than with WHO growth references (Table 2). In France, proportionally more children were classified as having height SDS below clinical cut-off values in all three indications with WHO vs. national growth references. In the UK, the proportion of children with GHD and TS who were classified as height SDS below –2 was the same as with the WHO growth references, whilst proportionally more children with SGA were classified as height SDS below clinical cut-off values with WHO vs. national growth references.

**Table 2:** Number and proportion of children with height SDS below clinical cut-off according to reference and difference and confidence limits for difference in sensitivity by indication and country for children aged ≥5 years.

**Growth hormone deficiency**				
	**n**_**n**_**/n**_**w**_**/N**	**National proportion below –2 SDS (%)**	**WHO proportion below –2 SDS (%)**	**Difference in sensitivity (%) (95% CI)**
Czech Republic	190/150/211	90	71	19 (14;24)
Denmark	97/73/110	88	66	22 (14;30)
France	537/675/853	63	79	–16 (–19;–14)
Germany	977/696/1192	82	58	24 (21;26)
Netherlands	22/16/23	96	70	26 (8;44)
Norway	46/32/54	85	59	26 (14;38)
Sweden	130/91/138	94	66	28 (21;36)
Switzerland	157/129/226	70	57	12 (8;17)
UK	73/73/99	74	74	0 (–3;3)
**Turner syndrome**				
	**n**_**n**_**/n**_**w**_**/N**	**National proportion below –2 SDS (%)**	**WHO proportion below –2 SDS (%)**	**Difference in sensitivity (%) (95% CI)**
Czech Republic	70/45/80	88	56	31 (21;41)
Denmark	31/22/35	89	63	26 (11;40)
France	80/93/144	56	65	–9 (–14;–4)
Germany	244/191/285	86	67	19 (14;23)
Netherlands	19/11/27	70	41	30 (12;47)
Norway	11/10/13	85	77	8 (–7;22)
Sweden	28/21/30	93	70	23 (8;38)
Switzerland	16/15/17	94	88	6 (–5;17)
UK	10/10/16	63	63	0 (0;0)
**Small for gestational age**				
	**n**_**n**_**/n**_**w**_**/N**	**National proportion below –2.5 SDS (%)**	**WHO proportion below –2.5 SDS (%)**	**Difference in sensitivity (%) (95% CI)**
Czech Republic	92/54/108	85	50	35 (26;44)
Denmark	32/22/40	80	55	25 (12;38)
France	209/224/299	70	75	–5 (–8;–2)
Germany	475/305/575	83	53	30 (26;33)
Netherlands	17/10/17	100	59	41 (18;65)
Norway	19/7/25	76	28	48 (28;68)
Sweden	24/8/25	96	32	64 (45;83)
Switzerland	14/9/17	82	53	29 (8;51)
UK	20/21/28	71	75	–4 (–10;3)

n_n_ = number of children with height below clinical cut-off values (national reference); n_w_ = number of children with height below clinical cut-off values (WHO reference); N = total; SDS, standard deviation score; WHO, World Health Organization.; Clinical cut-off values, below –2 SDS for growth hormone deficiency and Turner syndrome; –2.5 SDS for children with short stature born small for gestational age.

The difference in sensitivity varied between countries (Table 2). Analysis of pooled data from the seven countries with a higher classification rate (Czech Republic, Denmark, Germany, Netherlands, Norway, Sweden and Switzerland) based on the national growth references showed that relatively more patients (22% of children with GHD, 32% of children born SGA and 21% of children with TS), were classified with height below –2 (or –2.5) SDS using national growth references when compared with WHO growth references (*P*<0.0001 across all indications). The largest difference (national–WHO) for children with GHD was observed in Sweden (difference 28%; 94% vs. 66%; *P*<0.0001). For children with TS, the largest difference was observed in the Czech Republic (difference 31%; 88% vs. 56%; *P*<0.0001). Among short children born SGA, the most noticeable difference in the proportions of children aged ≥5 years with height SDS below –2.5 was observed in Sweden (difference 64%; 96% vs. 32%; *P*<0.0001).

### Children aged <5 years

As WHO growth standards are designed for use in children aged <5 years, this age group was analyzed for differences in height SDS between national growth charts and WHO growth standards. Despite low numbers of children aged <5 years in some countries, a similar pattern of proportionally more patients with height SDS below –2 (or –2.5 for SGA) was observed using national growth references compared with WHO growth standards across all indications, except in France and the UK (Table 3).

**Table 3:** Number and proportion of children with height SDS below clinical cut-off according to reference/standard and difference and confidence limits for difference in sensitivity by indication and country for all patients aged <5 years.

**Growth hormone deficiency**				
	**n**_**n**_**/n**_**w**_**/N**	**National proportion below –2 SDS (%)**	**WHO proportion below –2 SDS (%)**	**Difference in sensitivity (%) (95% CI)**
Czech Republic	69/62/80	86	78	9 (3;15)
Denmark	27/22/34	79	65	15 (3;27)
France	168/193/210	80	92	–12 (–16;–8)
Germany	229/214/260	88	82	6 (3;9)
Netherlands	11/9/14	79	64	14 (–4;33)
Norway	9/6/9	100	67	33 (3;64)
Sweden	39/37/41	95	90	5 (–2;11)
Switzerland	17/16/18	94	89	6 (–5;16)
UK	14/16/21	67	76	–10 (–22;3)
**Turner syndrome**				
	**n**_**n**_**/n**_**w**_**/N**	**National proportion below –2 SDS (%)**	**WHO proportion below –2 SDS (%)**	**Difference in sensitivity (%) (95% CI)**
Czech Republic	16/10/22	73	46	27 (9;46)
Denmark	4/3/5	80	60	20 (–15;55)
France	29/33/42	69	79	–10 (–18;–1)
Germany	65//60/77	84	78	6 (1;12)
Netherlands^†^	1/0/1	100	0	–
Norway^†^	2/2/4	50	50	–
Sweden^†^	2/2/3	67	67	–
Switzerland^†^	1/1/1	100	100	–
UK	8/9/11	73	82	–9 (–26;8)
**Small for gestational age**				
	**n**_**n**_**/n**_**w**_**/N**	**National proportion below –2.5 SDS (%)**	**WHO proportion below –2.5 SDS (%)**	**Difference in sensitivity (%) (95% CI)**
Czech Republic	56/51/58	97	88	9 (1;16)
Denmark	14/12/14	100	86	14 (–4;33)
France	120/135/144	83	94	–10 (–15;–5)
Germany	162/142/182	89	78	11 (6;16)
Netherlands	8/4/8	100	50	50 (15;85)
Norway	13/12/15	87	80	7 (–6;19)
Sweden	10/8/11	91	73	18 (–5;41)
Switzerland	7/5/8	88	63	25 (–5;55)
UK	15/16/16	94	100	–60 (–18;6)

n_n_ = number of children with height below clinical cut-off values (national reference); n_w_ = number of children with height below clinical cut-off values (WHO standard); N = total; SDS, standard deviation score; WHO, World Health Organization. Clinical cut-off values, below –2 SDS for growth hormone deficiency and Turner syndrome; –2.5 SDS for children born small for gestational age.

^†^Difference in sensitivity and 95% CI not shown due to low N (<5).

When pooling data from the seven countries with higher classification rates with national references vs. WHO growth standards, a significantly greater proportion of children with GHD had height SDS below –2 with national references (8%; *P*<0.0001). Among the children aged <5 years with TS, analysis of pooled data from the seven selected countries with higher classification rates revealed a significantly greater proportion of children with height below –2 SDS according to national growth references than with WHO growth standards (12%; *P* = 0.0003) (Table 3). Among children with SGA aged <5 years, 11% (*P*<0.0001) more patients were categorized with short stature using national growth references compared with WHO growth standards in Germany. Furthermore, analysis of pooled data from the seven selected countries with higher classification rates showed a significantly higher proportion with height below –2.5 SDS when evaluated with national vs. WHO growth standards (12%; *P*<0.0001).

## Discussion

We found significant differences across countries in the proportions of children with short stature and diagnosed with GHD, TS or born SGA using WHO growth standards/references compared with national growth references. Using WHO growth references, significantly fewer children aged ≥5 years with GHD (22%; *P*<0.0001), TS (21%; *P*<0.0001) or born SGA (32%; *P*<0.0001) in seven of the countries evaluated (Czech Republic, Denmark, Germany, Netherlands, Norway, Sweden and Switzerland) had height below clinical cut-off values than with the national growth references. In children aged <5 years, a significantly greater proportion of children with GHD (8%; *P*<0.0001), TS (12%; *P* = 0.0003) or born SGA (12%; *P*<0.0001) had height below clinical cut-off values with national growth references than with WHO growth standards in the same seven countries. The available national references used in this study range with respect to their time of construction from the French national reference collected from 1953 onwards and published in 1979 [9], to the Danish national reference that was published in 2014 [6]. Thus, one would expect that secular trends with increased average height over generations should reflect differences in cut-offs for short stature, strongly correlated with the date at which the reference was constructed. The results of this study show that the greatest differences from the WHO standards/reference were generally observed in those countries with the most recent published reference data; Czech Republic (2004) [23], Denmark (2014) [6], Germany (2001) [24], Norway (2013) [25], Sweden (2002) [26], Netherlands (2000) [7]. In France, which had the oldest national growth reference [9], a smaller proportion of children across all indications had height SDS below clinical cut-offs using national references than with the WHO growth standards/references. Our observation is consistent with a recent report by Scherdel *et al*., who documented that, with the exception of the first 6 months, the growth of French children up to 18 years of age and born between 1981 and 2007 was closer to that described by the WHO growth standards/references than to the French national reference used in our study [29].

Although the use of an appropriate growth standard/reference is important to detect short stature, growth charts are never ideal, especially as they are dependent upon accurate height measurements [30]. Moreover, it should be considered that growth charts form only a part of the clinical armory for detecting diseases or conditions with short stature [29].

Although WHO advocates the use of a single height-for-age chart worldwide [31, 32], several studies have demonstrated significant differences in height assessed using WHO and national height-for-age references [15, 28]. In a meta-analysis of data published in studies from 55 countries, involving over 11 million children aged <5 years, 20% of all values for mean height SDS were ≥0.5 SD from the means in WHO growth standards [33] and 44% of means for boys and 48% of means for girls were at least ±0.25 SD from corresponding mean values in WHO standards at four or more time points. Among outliers, Europeans were generally above 0.5 SD, and children from Saudi Arabia and Asian Indians were below –0.5 SD, suggesting inter-population differences that warrant consideration when evaluating patients with short stature [33]. In a large German study, Rosario *et al*. [15] demonstrated that mean heights for boys and girls aged ≤5 years were at the 60^th^ and 62^nd^ percentiles of WHO growth standards, respectively. On the basis of these findings, those authors recommended use of national growth curves over WHO growth standards [15]. Moreover, in Australia, Hughes *et al*. [34] documented that use of WHO growth standards to assess height was associated with consistent under-diagnosis of short stature, indicating that Australian children are taller than the WHO reference population.

Only a few studies have evaluated the impact of using WHO height standards/references compared with national references in pediatric diseases. Bonthuis *et al*. [35] found that 33% and 34% of 3402 children with end-stage renal disease from 13 European countries would be classified as short for age using WHO or Center for Disease Control and Prevention growth references, respectively, compared with 44% meeting the criteria using recent national or derived Northern and Southern European growth references. Likewise, Saari *et al*. [36] demonstrated a significantly (*P*≤0.001) higher sensitivity for detecting short stature among girls with TS using population-specific growth references than using WHO growth standards.

The secular trend in growth mandates regular updating of growth references and updated single-country height-for-age references can be assumed to provide optimal reference information. In the present analysis we have demonstrated a marked deviation from WHO growth standards/references for the countries with the most recently updated growth references (for example, Denmark and Norway [6, 25]), whereas France, using older growth references [9], classified a lower proportion of children with short stature than WHO standards/references. A key argument for the use of WHO growth standards is that breastfed children of non-smoking mothers represent a healthier population with a differential, healthier growth pattern. However, recent national cohorts including breastfed children of non-smoking mothers also showed increased height vs. WHO standards [6, 14].

As assessment of a child’s height in relation to the height distribution of the peer population is one of the best indicators of his or her general health and well-being [2], the use of outdated growth references may have significant implications for individual patients [37]. More children will meet the criteria for short stature when a reference population is taller than that used to develop WHO growth standards/references. Likewise, deviation from normal growth is underdiagnosed when outdated national references, such as those from France (1979) and the UK (1990), are used for the assessment of short stature.

The variations in height assessments in our study highlight the complexities of growth and growth monitoring. In addition to genetics, health, nutritional status, and psychosocial and environmental factors, differences in the rate of secular changes in growth between countries may all have an impact on population height [33].

Strengths of our study include the large sample size and the robustness of our findings across several European countries with updated growth references, even in children aged <5 years. Possible limitations may include lack of adjustment for ethnicity or immigrant populations, which may be important determinants of growth [38] and the lower numbers of children per indication aged <5 years, which restricted the use of statistical models for comparison of data on country level. In addition, data on the genetic composition of children with Turner syndrome were not available. However, descriptive data and analysis on pooled data indicated that the overall trends were the same as those described for the total patient cohort.

## Conclusions

The use of inappropriate growth standards/references to assess short European children may lead to significant reclassification to normal height, potentially delaying or leading to missed diagnosis of a growth disorder. This may prevent the timely identification of an underlying medical condition in children with short stature. Although the use of growth references may have limitations, mainly stemming from the secular trends in growth patterns and population differences, our data suggest that updated national or regional growth references may offer the most suitable option for monitoring the growth of European children with short stature.

### Ethics

NordiNet^®^ IOS is conducted in accordance with the Declaration of Helsinki; all patients provide written informed consent for data collection. All data collected within NordiNet^®^ IOS are anonymized. Approval of the local ethics committee or institutional review board at the individual center level in accordance with country-specific rules is a prerequisite for each center’s inclusion in NordiNet^®^ IOS.

### Clinical Trial Registration

NordiNet^®^ International Outcome Study is registered at ClinicalTrials.gov NCT00960128.
